# Preverbal Visual Assessment as an Early Neurodevelopmental Marker in Extremely Preterm Infants: Insights from the PremTEA Cohort

**DOI:** 10.1192/j.eurpsy.2026.10622

**Published:** 2026-07-31

**Authors:** A. Romero Teruel, S. Zeballos-Sarrato, E. Rodríguez-Toscano, M. Arriaga-Redondo, I. Pescador, J. Merchán-Naranjo, C. Polonio-Romero, L. Pina-Camacho, D. Blanco-Bravo

**Affiliations:** 1 Dr. Rodríguez Lafora Hospital; 2 Department of Neonatology; 3 Gregorio Marañón University General Hospital; 4 Department of Experimental Psychology, Cognitive Processes and Speech Therapy, Complutense University of Madrid; 5 CIBERSAM; 6 Institute of Psychiatry and Mental Health, Madrid, Spain

## Abstract

**Introduction:**

Extremely preterm infants (≤28 weeks GA) are at high risk for neurodevelopmental disorders, including ASD and ADHD. Early detection of atypical neurodevelopmental trajectories remains limited. The *Preverbal Visual Assessment* (PreViAs) questionnaire evaluates early visual behaviors across four domains: Visual Attention (VA), Visual Communication (VC), Visuomotor Coordination (VMC), and Visual Processing (VP).

**Objectives:**

1. Compare PreViAs scores between extremely preterm infants (EPI) and full-term newborns (FTN) at 12 and 24 months.

2. Examine associations between atypical PreViAs profiles and perinatal clinical variables.

3. Assess its potential as an early dimensional marker for neurodevelopmental risk.

**Methods:**

We conducted a prospective longitudinal cohort study including 58 EPI (GA <28 weeks) and 46 FTN recruited in the PremTEA study. PreViAs was administered at 12 and 24 months (corrected age for EPI). Clinical variables included intraventricular hemorrhage (IVH ≥ grade 3), periventricular leukomalacia (PVL), retinopathy of prematurity (ROP), and birth weight. Statistical analyses comprised Mann–Whitney U, Chi-square tests, and logistic regression models.

**Results:**

Global PreViAs scores were significantly lower in EPI than FTN at both 12 and 24 months (p<0.05). Notably, at 12 months, EPI showed deficits in VC, VMC, and VP domains; at 24 months, deficits persisted in VMC and VP. Furthermore, IVH ≥ grade 3 and cystic PVL were consistently associated with poorer outcomes across all domains (p<0.01). Longitudinal analysis revealed limited improvement in EPI, with persistent deficits in VMC (p=0.027) and VP (p=0.045).

**Conclusions:**

PreViAs enables early identification of atypical visual neurodevelopmental trajectories in high-risk infants. Detecting early visual dysfunction may support timely interventions aimed at improving neurodevelopmental outcomes in extremely preterm populations.

**Disclosure of Interest:**

None Declared

